# The Localisation of Missiles

**Published:** 1919-07-26

**Authors:** 


### WAR RADIOGRAPHY.

### The Localisation of Missiles.

Papers on this subject published in recent times do not tell us of any absolutely new methods, but there has been great ingenuity displayed in development and perfection of older procedures. We have long known the triangulation method which Mackenzie Davidson and Hedley first described in 1897, the application of the parallax principle as originally adapted by Levy-Dorn in 1898, and a number of forerunners of the Hirtz compass and similar directors. Even the idea of visualising the skin adjacent to foreign bodies by means of bismuth paste dates back to 1897, and on the whole one is probably justified in saying that long before the war every principle now in use had been fully described, and that during the last few years nothing has appeared beyond a number of improvements in apparatus and technique.

Nevertheless, there have been advances well worth recording and of considerable interest. It is beyond the scope of this article to describe in any detail the more complicated and perhaps the most accurate types of apparatus. Rather would one mention those procedures the chief value of which lies in their combined simplicity and relative accuracy, for the greatest need in war radiography has been that much of it should be possible with the assistance of a well-trained sister or orderly only, and that the whole of the *x*-ray work should be performed with as little obstruction and delay to the operator as possible. Many improvements towards this ideal have been made, and as such should be of great value to civil surgery of the future.

### Localisation in Limbs.

Two pieces of apparatus, described respectively by Cole and Hall-Edwards in 1917, are particularly useful when the foreign body is fixed in some portion of a limb. The mechanism and principle in both are practically identical. The limb is fixed to a base board, and attempt is made to keep up the initial, *i.e.* the radiological, position throughout any operative manipulations. At the sides of the base board metal uprights are fixed carrying metal pointers, which can be directed exactly towards the foreign body by screening, the guides then fixed for line and the pointers removed or replaced at times as the surgeon wishes. The *direction* of each is maintained throughout and the intersection of their lines gives a good and practical guide to the site of a missile. The method has considerable value, as the lateral supports do not obstruct the operator, and in practice the eye can readily follow the indication of the pointers when their ends are brought reasonably near to the limb or incision. This instance is also of interest in connection with the vexed question of operating under direct *x*-ray control, as one screening only is necessary, provided the limb, base board, and uprights do not alter their relative position afterwards.

### A Localising Couch.

In 1918 Mackenzie Davidson described a localising couch which, although not adapted for direct surgical work, combines accuracy of localisation with both simplicity in reading and small amount of mechanical manipulation. The foreign body is localised from below up, and the tube, contained in a box below the table, is attached by a single connecting arm and works in unison with the screen above the limb. They are fixed at a constant distance of 50cm., and, using the usual method of triangulation, this fixed anode to screen measurement makes it possible to read off the depth directly from a scale without any mathematical calculations. An ingenious arrangement of the lead plate diaphragm makes it possible in each position to view the foreign body in a small area of illumination—a valuable aid to accuracy when dealing with small fragments. This couch provides a most happy combination of speed and accuracy, but an even quicker and more adaptable production has been in extensive use with the Italian Army, and first described in 1917. The tube and screen form a complete mobile unit, their distance is fixed, and they rotate together at the ends of a diameter. The shadow of a foreign body falls on this diameter, and the apparatus is raised or lowered until this shadow is immobile on the screen during rotation. The foreign body must then lie exactly at the centre, *i.e.*, midway between the tube and screen, and by a graduated pointer the depth from any skin surface can be read off instantly.

### A Method without Apparatus.

Shenton in 1918 published details of an extremely simple method in which no calculations of any kind are needed. If one gives the main points the reader can construct most of the details for himself. Two plates are placed over the patient, one above the other, film side down, at 10cm. apart. The tube distance does not matter. Two exposures are made, the second after laterally moving the tube a short distance, amount immaterial. Double shadows of the foreign body appear on each plate, those on the upper plate being further apart than those on the lower. These displacements are measured, marked off on two parallel lines, and drawn 10cm. apart at right angles to the edge of a piece of paper. The line obtained by joining the inner ends of these two marked-off portions is continued downwards to intersect the edge of the paper, and the point where it does so graphically represents the actual position and depth of the foreign body below the plates. The method can be used with the simplest *x*-ray equipment, and is extremely valuable in practice, it being possible to dispense with plates and use a screen, provided distances are kept with reasonable accuracy.

**War Radiography—(*continued*).**

And this brings us to a point which, although applying chiefly to evacuation units, is of great importance in civil practice when we aim at *rapid* work, sufficiently rapid to be done in actual conjunction with surgical removal. For intricate or obscure cases complicated apparatus, extreme accuracy, and plates are essential, but for most of the urgent work the screen only is possible. Hence the importance and value of the last two methods, and one gives them in this brief outline as examples of procedures which may come into very general use. Most of the recent literature on radiology deals, however, with further and extreme refinement of mechanical methods and instruments. These we must leave at the mere reference; they are rightly confined to the experienced hands of the specialist, but nevertheless the war has given us a number of simplified methods, possible of extensive employment by those surgeons who would develop the principle of operating when necessary under immediate screen control. It is a point which those interested in development and modernisation of any surgical clinic may well bear in mind. *X*-rays in the theatre have many possible uses, and we now have several recent simplifications which go some distance towards removing the old-time objections.

